# Effects of Extra-Long-Acting Recombinant Bovine FSH (bscrFSH) on Cattle Superovulation

**DOI:** 10.3390/ani12020153

**Published:** 2022-01-09

**Authors:** Miguel A. Gutiérrez-Reinoso, Constanza J. Aguilera, Felipe Navarrete, Joel Cabezas, Fidel O. Castro, Ignacio Cabezas, Oliberto Sánchez, Manuel García-Herreros, Lleretny Rodríguez-Alvarez

**Affiliations:** 1 Laboratorio de Biotecnología Animal, Departamento de Ciencia Animal, Facultad de Ciencias Veterinarias, Universidad de Concepción (UdeC), Chillán 3780000, Chile; mgutierrezreinoso@hotmail.com (M.A.G.-R.); consaguilera@udec.cl (C.J.A.); fenavarr@udec.cl (F.N.); joelcabezas@gmail.com (J.C.); fidcastro@udec.cl (F.O.C.); 2 Facultad de Ciencias Agropecuarias y Recursos Naturales, Carrera de Medicina Veterinaria, Universidad Técnica de Cotopaxi (UTC), Latacunga 050150, Ecuador; 3 Departamento de Ciencias Clínicas, Facultad de Ciencias Veterinarias, Universidad de Concepción (UdeC), Chillán 3780000, Chile; oscabeza@udec.cl; 4 Departamento de Farmacología, Facultad de Ciencias Biológicas, Universidad de Concepción, Victor Lamas 1290, Concepcion 4070386, Chile; osanchez@udec.cl; 5 Instituto Nacional de Investigação Agrária e Veterinária (INIAV), 2005-048 Santarém, Portugal

**Keywords:** recombinant FSH, superovulation, ovarian function, embryo production, gene expression, cattle

## Abstract

**Simple Summary:**

While the clinical effectiveness of medium-acting bovine follicle stimulating hormone (bFSH) is well-established for superovulation (SOV) protocols in cattle, the use of long-acting bovine recombinant follicle stimulating hormone (brFSH) remains the golden target. The application of a bFSH with prolonged half-life could improve substantially the results obtained regarding the ovarian superstimulation response and the quality and quantity of the embryos produced. Moreover, the management schemes may be improved by reducing the number of FSH applications needed during the SOV protocol. The development of a new extra-long-acting bovine single-chain recombinant follicle stimulating hormone (bscrFSH) provides interesting advantages to the SOV protocols as the present study shows, by improving the results obtained compared to the use of the conventional follicle stimulating hormone from purified pig pituitary extract (NIH-FSH-p).

**Abstract:**

Over the last few years, several commercial FSH products have been developed for cattle superovulation (SOV) purposes in Multiple Ovulation and Embryo Transfer (MOET) programs. The SOV response is highly variable among individuals and remains one of the main limiting factors in obtaining a profitable number of transferable embryos. In this study, follicle stimulating hormone (FSH) from different origins was included in two SOV protocols, (a) FSH from purified pig pituitary extract (NIH-FSH-p; two doses/day, 12 h apart, four consecutive days); and (b) extra-long-acting bovine recombinant FSH (bscrFSH; a single dose/day, four consecutive days), to test the effects of bscrFSH on the ovarian response, hormone profile levels, in vivo embryo production and the pluripotency gene expression of the obtained embryos. A total of 68 healthy primiparous red Angus cows (*Bos taurus*) were randomly distributed into two experimental groups (*n* = 34 each). Blood sample collection for progesterone (P4) and cortisol (C) level determination was performed together with ultrasonographic assessment for ovarian size, follicles (FL) and corpora lutea (CL) quantification in each SOV protocol (Day 0, 4, 8, and 15). Moreover, FSH profiles were monitorised throughout both protocols (Day 0, 4, 5, 6, 7, 8, 9, 10, and 15). In vivo embryo quantity and quality (total structures, morulae, blastocysts, viable, degenerated and blocked embryos) were recorded in each SOV protocol. Finally, embryo quality in both protocols was assessed by the analysis of the expression level of crucial genes for early embryo development (*OCT4, IFNt, CDX2, BCL2*, and *BAX*). P4 and cortisol concentration peaks in both SOV protocols were obtained on Day 15 and Day 8, respectively, which were statistically different compared to the other time-points (*p* < 0.05). Ovarian dimensions increased from Day 0 to Day 15 irrespective of the SOV protocol considered (*p* < 0.05). Significant changes in CL number were observed over time till Day 15 irrespective of the SOV protocol applied (*p* < 0.05), being non- significantly different between SOV protocols within each time-point (*p* > 0.05). The number of CL was higher on Day 15 in the bscrFSH group compared to the NIH-FSH-*p* group (*p* < 0.05). The number of embryonic structures recovered was higher in the bscrFSH group (*p* = 0.025), probably as a result of a tendency towards a greater number of follicles developed compared to the NIH-FSH-p group. *IFNt* and *BAX* were overexpressed in embryos from the bscrFSH group (*p* < 0.05), with a fold change of 16 and 1.3, respectively. However, no statistical differences were detected regarding the *OCT4, CDX2, BCL2,* and *BCL2/BAX* expression ratio (*p* > 0.05). In conclusion, including bscrFSH in SOV protocols could be an important alternative by reducing the number of applications and offering an improved ovarian response together with better embryo quality and superior performance in embryo production compared to NIH-FSH-p SOV protocols.

## 1. Introduction

The Assisted reproductive technologies (ARTs) have revolutionised dairy and meat production systems in the global industry. ARTs includes different techniques, such as: (a) low complexity (Artificial Insemination (AI) and Multiple Ovulation Embryo Transfer (MOET)) and (b) high complexity (in vitro Embryo Production (IVP), Intracytoplasmic Sperm Injection (ICSI), Cloning and Parthenogenetic Embryo Production (PP)). From the early 1940s until the 1970s, embryo transfer in cattle was carried out by using pregnant mare serum gonadotropin (PMSG) and follicle-stimulating hormone (FSH) [1]. From the 1970s onwards, the use of commercial pituitary extracts and prostaglandins was developed, and later, in the 1980s and 1990s, partially purified pituitary extracts and progesterone devices established commercial embryo transfer (ET) activities [2,3,4,5]. Although ovum pick up (OPU) and IVP are currently practised on a large scale, superovulation techniques have been the main tools for embryo production in cattle for decades, with uterine lavage collection being a very effective technique for embryo production, with an increased viability and cryotolerance [6]. The development of new techniques and protocols for ovarian superstimulation in bovine has significantly improved with regard to synchronisation and hormonal protocols [1,4]. However, the average number of embryos produced per donor (quantity) has not improved, but there has been an advancement regarding the number of transferable embryos (quality) recovered per unit of time [4]. The embryo quality in ET programs is directly influenced by donor superovulatory treatment [7]. However, there is still a high degree of inefficiency in the superovulatory response due to the variability of pituitary extracts, among other factors [6,8,9], in relation to gonadotropins’ (FSH and LH) content and ratio, which has not been improved and has affected the cost-effectiveness of commercial bovine embryo transfer. Thus, the superovulatory response remains one of the main problems to be solved [10,11]. Adequate FSH levels and the absence of LH improve the superovulatory response, oocyte quality, ovulation rate, and the number of embryos collected [12,13]. However, other authors argue that low LH concentrations are necessary for successful superovulation [4]. Protocols are currently based on the exclusive use of commercial porcine and ovine pituitary extracts [14,15]. Recently, a study has been developed by using recombinant ovine FSH (roFSH) performing superovulation in sheep and cattle with similar results compared to conventional treatments [7]. Despite the above report there is very little information on the use of a pure recombinant bovine FSH technology that could generate high superovulatory responses and better embryo quality compared to conventional FSH [16,17,18].

In cattle, gene expression changes during the transition from embryo to conceptus are not well elucidated. Differences in the expression of genes associated with pluripotency and IFN-tau have been found for all transcripts between blastogenesis and elongation [19]. In bovine, the regulation of gene expression in the pre- and peri-implantation embryo is crucial to ensure its correct development [19,20]. These events are regulated by epigenetic mechanisms that include chromatin remodelling, changes in histone acetylation patterns and DNA methylation [21]. These epigenetic modifications remodel chromatin and provide access to transcription regulatory factors for control of gene expression [22]. In bovine, there are variations in the embryo gene expression associated with pluripotency as a result of ARTs such as cloning and IVP [19]. Therefore, conventional gonadotropins used in superstimulation protocols could also generate variations in gene expression and this could be associated with a decrease in embryo competence and/or embryo quality [23]. Thus, the different origins of gonadotropins (e.g., recombinant FSH) could determine different levels of gene expression linking blastocyst-conceptus transition processes.

Therefore, the aim of the present study was to determine whether the administration of a single daily dose of bovine recombinant gonadotropin (bscrFSH—with extra-long-acting recombinant technology by additional N-glycosylation) could generate a supeovulatory response comparable or superior to conventional protocols in cattle. Furthermore, our aim was to evaluate embryo production and quality, embryo morphology and pluripotency gene expression in embryos obtained by using a single-dose per day of recombinant bovine gonadotropin (bscrFSH) protocol compared to those obtained by a conventional double-dose per day pituitary-derived gonadotropin (NIH-FSH-p) superovulation protocol.

## 2. Materials and Methods

### 2.1. Ethics Statement

The authors declare that the present study was conducted according to the following Ethics′ Code for animal experiments as reflected in the ARRIVE guidelines available at http://www.nc3rs.org.uk/ARRIVEchecklist (Accessed Date: 7 July 2020). This study was approved by the Bioethics Committee for the use of experimental animals at the Universidad de Concepción—Campus, Chillán—Chile (Approval Date: 1 September 2018, Code Number: CBE-8269/2018).

### 2.2. Reagents and Media

All reagents used in this study, unless otherwise stated, were of analytical grade and were purchased from Sigma-Aldrich Chemical Company (Chile). All media were prepared on the day of use and adapted to bovine species.

### 2.3. Geographical Location

The present study was conducted at the facilities of the Veterinary Medicine Faculty, Livestock Sciences Section, University of Concepción—Campus Chillan (Ñuble, Chile), southern zone 36°36′24″ S 72°06′12″ W. This region has annual temperatures fluctuating between 13.5 °C and 14 °C, with maximum values in the hottest month (January) of 38 °C and an average minimum in the coldest month (July) between −2 °C and 5 °C. Annual rainfall is 1058 mm. and average altitude is 124 m.a.s.l. The study was conducted between July 2019 and November 2021.

### 2.4. Single-Chain Recombinant Bovine FSH (bscrFSH) Hormone Synthesis and Composition

bcsrFSH is a biobetter variant of bovine FSH. A single-chain recombinant bovine FSH (bscrFSH) gene sequence variant was designed considering the *Bos taurus* alpha and beta subunits linked through a flexible spacer peptide with two potential N-glycosylation sites. According to its amino acid sequence, bcsrFSH has a predicted molecular weight of 26.6 kDa. However, the 6 N-glycans attached to the molecule add an additional 24 to 30 kDa. Thus, bcsrFSH is a 51 to 57 kDa glycoprotein, where approximately 50% of the molecular weight is provided by oligosaccharides. The identification and purification processes were based on a 6-residue histidine tag which was incorporated at the C-terminal end. The lentiviral vector was transduced into the CHO (suspension culture in Chinese Hamster Ovary cell) cell line in an animal-protein-free culture medium. The bscrFSH hormone was purified by affinity chromatography, which guarantees a purity level of over 97% (total absence of LH hormone), ensuring high activity, potency, homogeneity, and reproducibility of the results obtained among batches. These modifications increase both, the hormone stability and the half-life in the organism (extra-long-acting hormone based on preliminary trials which suggested circulating half-life higher than 48 h). The product was lyophilised to maintain potency under normal storage conditions. The current prototype of bscrFSH comes as a box containing two vials. One of them contains 0.3 mg of lyophilised bscrFSH, equivalent to 300 µg total, while the second one contains 20 mL of reconstitution solution. Once reconstituted, the concentration remains in 15 µg/mL. The bscrFSH reconstitution solution is not compatible with the reconstitution solution from commercial hormones. The specific activity of bscrFSH has been calibrated against the first international standard for FSH (92/642). The vehicle contains a stabilising and bacteriostatic aqueous solution containing sucrose, sodium citrate dihydrate, m-cresol, DL-methionine, polysorbate 20, and purified water q.s.c. which stabilise the hormone and maintain its activity for at least one month after reconstitution. The molecule bscrFSH has been protected by patent application with a priority number CL20181347 and the application number in Europe is EP19733942.7.

### 2.5. Experimental Groups and Superovulation (SOV) Protocols

A total of 68 healthy primiparous red Angus cows (*Bos taurus taurus*), (Day ~75 post-partum, Body weight: 450 ± 50 kg; Age 3 ± 0.5 y.o., Body condition score: 3.5 ± 0.5) were included in the study. The animals were exposed to the same environmental, nutritional (water, mineralised salt, and ad libitum grazing) and management conditions. The individuals were randomly distributed into two experimental groups: control group (NIH-FSH-p: follicle-stimulating hormone from Purified Pig Pituitary Extract; *n* = 34), and treatment group (bscrFSH: bovine single-chain recombinant follicle-stimulating hormone; *n* = 34). Regarding the NIH-FSH-p superstimulation group, a conventional protocol was applied (two doses daily in decreasing amounts each day over 4 days) and, regarding the bscrFSH group, a conventional protocol was applied with a modification in the application interval between doses (24 h. between injections/decreasing dose). In both groups, the total doses were calculated according to the total dose recommended for dairy cattle (100%): in beef cattle, this was between 50 and 75% of the total dose; and in heifers, this was 80% of the total dose (depending on the breed considered) [24]. For in vivo embryo production, the superovulation conventional procedure, insemination and embryo collection methods described by [25] were performed for the control group and the frequency of application of bscrFSH was modified for the treatment group.

Treatments in control and treatment groups (superovulation protocols) started at 08:00 a.m. regardless of follicular or luteal phase.

**Figure 1:**
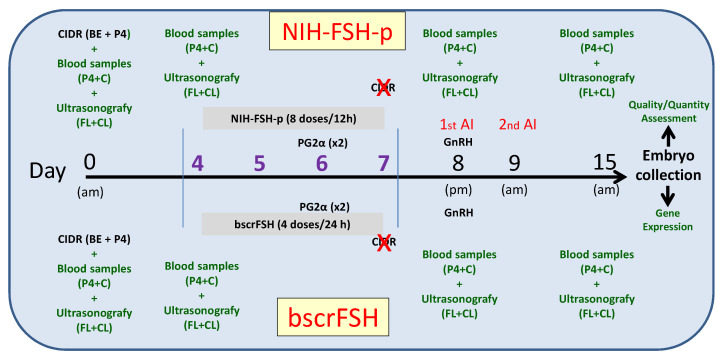
Experimental design including the both superovulation (SOV) protocols (NIH-FSH-p and bscrFSH). NIH-FSH-p: 280 mg (total); 2 applications per day (a.m./p.m.) scheduled at 12 h intervals for four days (Day 4, 5, 6 and 7; 50-50, 40-40, 30-30, 20-20 mg i.m., respectively; 8 applications total). bscrFSH: 170 µg (total); a single application per day (a.m.) scheduled at 24 h intervals for four days (Day 4, 5, 6 and 7; 55, 50, 40, and 25 µg i.m. respectively; 4 applications total). Blood samples for FSH determination were obtained thoroughly in each SOV protocol (Day 0, 4, 5, 6, 7, 8, 9, 10, and 15).

In the NIH-FSH-p group protocol, Day 0 was considered as the time of intravaginal P4 progesterone releasing intravaginal device application (CIDR-1.38 gr) + 2.5 mg intramuscular (i.m.) estradiol benzoate BE2 + 100 mg intramuscular (i.m.) progesterone P4 [8,9,26]. On Day 4 post-CIDR application, the superstimulation protocol was started by NIH-FSH-p application. Control group received 280 mg NIH-FSH-p (Folltropin-V; Bioniche Animal Health, Belleville, Ontario, Canada) divided into eight decreasing doses, with two applications per day in an a.m./p.m. schedule at 12 h intervals for four days (50-50, 40-40, 30-30, 20-20 mg i.m.). In parallel with the fifth and sixth NIH-FSH-p application, two doses of PGF2α alpha (500 µg D-cloprostenol each i.m.) were administered, and additionally, the intravaginal device was removed, giving, at the same time, the seventh NIH-FSH-p injection [27]. Forty-eight hours after the first dose of PGF2α alpha (Day 8 of the protocol a.m.) the donors presented oestrus, and 12 h later (p.m.), the first insemination was performed together with 0.008 mg buserelin acetate (GnRH) administration (i.m.). The second insemination was performed 48 h after oestrus (Day 9 of the protocol) [27]. The embryos were collected 7 days after the oestrus onset (Day 15 of the protocol), by trans-rectal uterine lavage (closed system) [28]. The schematic design of the experimental control group (NIH-FSH-p) can be seen in Figure 1.

In the bscrFSH group protocol, Day 0 was considered as the time of intravaginal P4 progesterone releasing intravaginal device application (CIDR-1.38 gr) + 2.5 mg intramuscular (i.m.) estradiol benzoate BE2 + 100 mg intramuscular (i.m.) progesterone P4 [8,9,26]. On Day 4 post-CIDR application, the supestimulation protocol started with the FSH-p application. Treatment group received 170 µg bscrFSH (Cebitropin B, Centro de Biotecnologia y Biomedicina Spa, Concepción, Chile) divided into four decreasing doses, distributed in a single application per day in a.m, scheduled for four days (55, 50, 40, and 25 µg i.m.). In parallel with the third bscrFSH application, two PGF2α alpha doses (500 µg D-cloprostenol i.m. each) were administered. Additionally, the intravaginal device was removed simultaneously with the fourth bscrFSH application. Moreover, 48 h after the first PGF2α alpha dose (day 8 of protocol a.m.) the donors manifested oestrus, and 12 h. later (p.m.) the first insemination was performed together with 0.008 mg buserelin acetate (GnRH; i.m.) administration. The second insemination was carried out 24 h. after oestrus (day 9 of the protocol a.m.) [27]. The embryos were collected at Day 7 after the oestrus onset (day 15 of the protocol), by trans-rectal uterine lavage (closed system) [28]. The schematic design of the experimental treatment group (bscrFSH) is represented in Figure 1.

### 2.6. Ultrasonographic Assessment

A reproductive evaluation was performed before starting the superstimulation protocols by using transrectal ultrasonography (Sonoscape X3Vet; 7.5 MHz sector translator probe, China) to check ovarian cyclicity (corpus luteum presence or absence) and uterine status. In addition, the animals were classified according to the number of follicles (range 3 to 7 mm) or by the presence of a dominant follicle or corpus luteum. If necessary, a corrective drug treatment was applied to stimulate cyclicity in the donors (Progesterone P4—Gonadotropins GnRH—Prostaglandin PGF2α) [29]. The evaluation of control and treatment group donors was performed by ultrasonography on the day of embryo collection (Day 15 of the protocol). The diameter of the ovaries (left and right), diameter and number of follicles and corpora lutea were determined in each individual (Day 0: CIDR device application; Day 4: first FSH application; Day 8: oestrus and Day 15: embryo collection).

### 2.7. Blood Sampling and Hormonal Analyses

The sandwich Enzyme-Linked Immunosorbent Assay (ELISA) was carried out for bscrFSH half-life determination. The ELISA protocol was developed and validated for bovine blood plasma samples in our laboratory. Blood samples were collected from control and treatment individuals before, during and after the superovulation protocol for bscrFSH concentration determination; Day 0 (CIDR application), Day 4, Day 5, Day 6, Day 7, Day 8 (oestrus), Day 10 and Day 15 (embryo collection). Plasma concentrations of bscrFSH were determined using the ELISA protocol as follows; briefly, 100 µL of monoclonal anti-bscrFSH mouse antibody (10 µg/mL) was added for bscrFSH detection (incubation overnight at 4 °C). The ELISA plates were washed once by using Phosphate Buffered Saline (PBS/Tween 0.05%). Then, the wells were blocked by adding 300 µL of skim milk (3%) in PBS for 1 h. at 37 °C. After this, the plates were washed twice by using PBS/Tween 0.05%. Then, 100 µL/well was added for the bFSH standard curve (312, 156, 78, 39, 19, 9.7, 4.9, 2.4, 1.2, 0.6 ng/mL in bovine serum) and samples were incubated for 2 h. at 37°C to be analyzed (duplicate). After this, the plates were washed three times by using PBS/Tween 0.05%. Then, 100 µL of anti-bscrFSH chicken serum antibody (1% in skim milk) was added in PBS/Tween 0.05% at 1:40,000 (*v/v*) dilution, and then were incubated for 1 h at 37 °C. After that, the plates were washed four times by using PBS/Tween 0.05%. Then, 100 µL/well of anti-IgY antibody (1% in skim milk) was added in PBS/Tween 0.05% at 1/20,000 (*v/v*) dilution, and then was incubated for 1 h at 37 °C. Once again, the plates were washed four times by using PBS/Tween 0.05%. Finally, 100 µL/well of OPD solution (25 mL substrate buffer + 10 mg OPD + 10 µL H_2_O_2_) was added and incubated in the dark for 10 min. The incubation was stopped by adding 2.5 M H_2_SO_4_ (50 µL/well). The ELISA plates were read at a wavelength of 492 nm. Moreover, blood samples were collected from control and treatment individuals during the superovulation protocol for progesterone (P4) and cortisol (C) concentration determination and on Day 0 (CIDR application), Day 8 (oestrus), and Day 15 (embryo collection). The quantification of plasma C and P4 concentration was performed at the Laboratory of Animal Physiology and Endocrinology (University of Concepción, UdeC) by using radioimmunoassay (RIA) kits for C and P4, respectively (CORTISOL-RIA-CT (KIPI28000) and PROG-RIA-CT (KIP1458) DIASource ImmunoAssays, SA Louvain-la-Neuve, Belgium), previously validated for bovine species. The intra-assay coefficient of variation for cortisol quantification was 0.51–1.49% and for progesterone was 0.76–3.75%.

### 2.8. Embryo Quality and Quantity Assessment

First, the morphological assessment of all the structures collected per donor from control and treatment was carried out. A classification of viable embryos, non-viable- degenerated embryos and unfertilised oocytes (UFOs) was performed. The selection of viable embryos allowed us to classify them into morulae and grade I and II blastocysts. The criteria for morphological embryo classification were implemented according to the guidelines of the International Embryo Technology Society (IETS; [30]), based on two criteria: (1) quality (Grade I, II and III) and (2) developmental stage (morula, early blastocyst and blastocyst).

### 2.9. Primer Design and PCR Protocol

All analysed embryos belonged to the same developmental stage (blastocyst). Embryos were randomly selected within each experimental group. At least one embryo for every three cows was randomly selected. Reverse Transcription (RT) was performed with the Cells-to-cDNA^TM^ II Kit, Reverse Transcription without RNA Isolation (Thermofisher, AM1723, Burlington, ON, Canada), according to the manufacturer’s instructions. Briefly, 20 µL of lysis buffer was added to each embryo and was incubated at 75 °C for 10 min. Then, 1 µL DNase I was added and incubated at 37 °C for 15 min. DNase was then inactivated at 75 °C for 5 min. Mix 1 and mix 2, consisting of the reaction additives dNTP mix, first strand primer, 10XRT buffer, M-MLV and RNase inhibitor, were added and incubated at 42 °C for 60 min. After this, it was incubated at 95 °C for 10 min to inactivate RT. Then, cDNA was ready and PCR with ddCt was performed. The PCR reaction was performed by PCR mix by using the KiCqStart^®^ SYBR^®^ Green qPCR ReadyMix™ enzyme (Sigma-Aldrich, KSPQ12012G, St. Louis, MO, USA). Each PCR was performed on a QPCR Mx3005P Real-Time PCR System (Agilent) configured as follows: 1 cycle at 95 °C for 30 s, 40 cycles of 95 °C for 5 s. and an annealing temperature of 55 °C for 15 s, 72 °C for 10 s and a final extension of 65 °C for 30 s. The primers designed are represented in Table 1.

### 2.10. Statistical Analyses

Statistical analyses were conducted by using SPPS^®^ v.25 package software (SPSS Statistics for Windows, v. 25.0: IBM Corp., Chicago, IL, USA). The results obtained from the samples were analysed running paired *t*-test, one-way ANOVA and as a post-hoc analysis Tukey test. The gene expression results were analysed by using Wilcoxon’s Rank Sum test. FSH, P4 and C results were analysed by repeated measures in the general linear model previously checked for normality and homogeneity determination by using Shapiro- Wilk and Levene tests. In order to compare the two group means, Student’s *t*-test was carried out. When the parametric test prerequisite was not obtained, the Mann- Whitney *U* test was performed. Finally, the Bonferroni post-hoc correction test was applied in multiple comparisons. Statistical significance was set as *p* < 0.05.

## 3. Results

### 3.1. Determination of Recombinant Bovine Single-Chain FSH (bscrFSH) Half-Life over Time during the SOV Protocol

**Figure 2:**
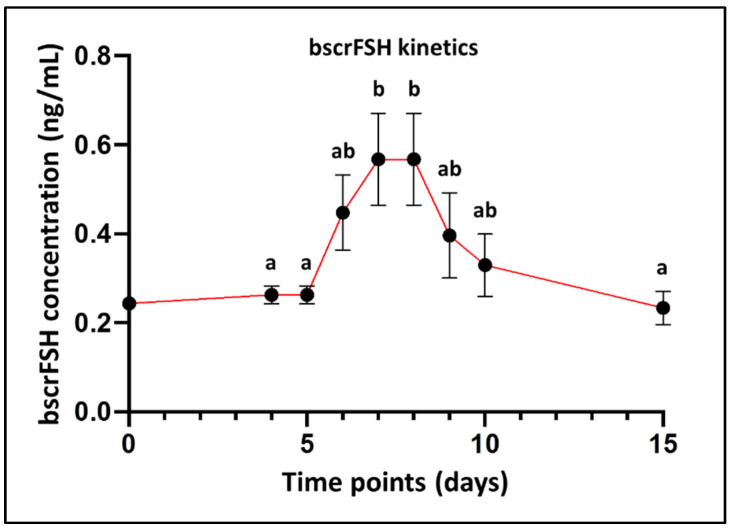
Bovine single-chain recombinant FSH (bscrFSH) half-life over time during the SOV protocol. Plasma bscrFSH concentrations till Day 15 after intramuscular (i.m.) administration of bscrFSH at 170 µg total dose distributed in a single application per day, four-days schedule (55, 50, 40, and 25 µg/day), in post-partum cows (*n* = 7). Values (Mean ± S.E.M.) with different superscript letters (a,b) between time points are significantly different (*p* < 0.05).

The bscrFSH concentrations after the administration of bscrFSH in a single application per day, divided into four decreasing doses (four-days schedule; 55, 50, 40, and 25 µg/day, respectively) in post-partum cows are represented in Figure 2. After four decreasing administrations of bscrFSH, the mean concentration increased significantly over time till Day 7 (*p* < 0.01). However, the bscrFSH concentration peak was maintained at similar levels from Day 7 to Day 8 (*p* > 0.05). Statistically significant differences were observed when Day 7 and Day 8 were compared to other time points (Day 4, 5, and 15; *p* < 0.05).

### 3.2. Ovarian Response: Ovary Dimensions, Follicles and Corpora Lutea Evaluation over Time; NIH-FSH-p vs. bscrFSH-Derived SOV Protocol Application

Ovarian dimensions increased from Day 0 to Day 15 (*p* < 0.001) but did not differ between ovaries within time-points, and there were no interactions (*p* > 0.05); therefore, data from the left and right ovaries were pooled. Ovarian length increased from Day 0 to Day 15 of the SOV protocol (*p* < 0.05; Table 2). Ovarian width increased as well from Day 0 to Day 15, irrespective of the SOV protocol considered (*p* < 0.05). No significant differences were observed regarding ovarian length or width within the same day between both SOV treatments (*p* > 0.05; Table 2).

**Figure 3:**
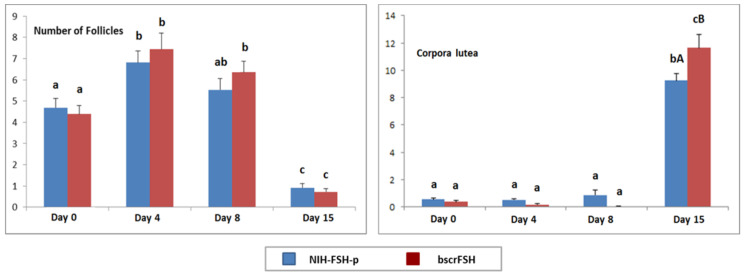
Ovarian structures (follicles and corpora lutea) over time; NIH-FSH-p vs. bscrFSH SOV protocol. The results are presented as Mean ± S.E.M. Bars (Mean ± S.E.M.) with different superscripts (a–c) denote statistically significant differences between time-points (days) (*p* < 0.05). Values (Mean ± S.E.M.) with different superscript letters (A,B) within each time-point (day) are significantly different between SOV protocols (*p* < 0.05). Unit: ng/mL.

The number of follicles tended to increase from Day 0 to Day 8, irrespective of the SOV applied (*p* < 0.05; Figure 3); however, the total number of follicles was not significantly different when the NIH-FSH-p and bscrFSH SOV protocols were compared, irrespective of the time-point considered (*p* > 0.05; Figure 3). The right and left ovaries did not differ for the overall number of follicles (*p* > 0.05). Although the number of ovarian follicles changed over time (*p* < 0.05 Figure 3), there were no interactions between SOV protocol and left or right ovary, indicating that the pattern of ovarian follicle production based on different SOV protocols did not differ between the right and left ovary (*p* > 0.05). Therefore, data for the left and right ovaries were pooled. Maximum follicle production increased at Day 4 and Day 8 (*p* < 0.05; Figure 3). Thus, the number of follicles in the ovaries showed significant changes over time till Day 15, irrespective of the SOV protocol applied (*p* < 0.05; Figure 3).

The number of corpora lutea regarding each SOV protocol applied are shown in Figure 3. There was no effect on CL production between ovaries (*p* > 0.05). Thus, no interactions between SOV protocol and left or right ovary were observed, indicating that the pattern of CL production based on different SOV protocols did not differ between the right and left ovary (*p* > 0.05). Therefore, data for the left and right ovaries were pooled (Figure 3). Maximum CL production was obtained at Day 15; moreover, significant differences were observed regarding the number of CL in the ovaries at Day 15 between SOV protocols, being higher in the bscrFSH group (*p* < 0.05; Figure 3).

### 3.3. Hormone Level Profiles during NIH-FSH-p vs. bscrFSH-Derived SOV Protocol Application: Concentrations of P4 and Cortisol over Time

Before CIDR application (Day 0), the plasma concentration of P4 did not exceed ~4 ng/mL but varied in the following time-points (Table 3). On the other hand, cortisol concentrations were less variable than those of P4 among and within time-points and groups and were between ~4 and ~12 ng/mL. There was a discharge of both hormones; however, the discharge was carried out at different time-points. As expected, the maximum P4 and cortisol plasma concentrations were obtained on Day 15 and Day 8, respectively (since the beginning of the SOV protocol), being 61.27 ± 7.35 and 74.13 ± 8.93 ng/mL for P4 in the NIH-FSH-p and bscrFSH-derived SOV protocol, respectively (*p* < 0.05), and 13.14 ± 4.88 and 12.51 ± 3.91 ng/mL for cortisol in the NIH-FSH-p and bscrFSH-derived SOV protocol, respectively (*p* > 0.05; Table 3). The time between the cortisol and P4 peaks differed by more than 7 days for any one animal. After the cortisol peak, the concentrations fell to levels similar to those observed during the first 4 days for any one SOV treatment (*p* > 0.05).

Statistically significant differences were detected regarding P4 concentration levels between Day 15 and the rest of the time-points evaluated (Days 0, 4, and 8; *p* < 0.05) as well as between cortisol concentrations on Day 8 and any other time-points studied (Days 0, 4, and 15; *p* < 0.05), irrespective of the SOV protocol analysed. No statistically significant differences were observed within the rest of the time-points for P4 or cortisol plasma levels (*p* > 0.05; Table 3).

### 3.4. In Vivo Embryo Quantity and Quality after NIH-FSH-p and bscrFSH-Derived SOV Protocol Application

The total structure collection efficiency (number of different embryonic structures recovered at different stages) differed between SOV protocols (*p* < 0.05; Table 4). The number of structures recovered tended to be greater in the bscrFSH protocol group (*p* = 0.025), probably as a result of a tendency towards a greater number of follicles developed compared to the NIH-FSH-p group (Figure 3).

The number of viable (grade I) embryos collected was statistically lower in the NIH-FSH-p group compared to the bscrFSH protocol group (*p* = 0.01). Moreover, the majority of embryonic structures collected in the bscrFSH protocol group were morulae, being statistically higher compared to the NIH-FSH-p group (*p* = 0.007). Regarding the early blastocysts and blastocysts parameters no statistical differences were observed between SOV protocols (*p* > 0.05). The NIH-FSH-p group had a greater number of degenerated structures compared to the bscrFSH protocol group (*p* = 0.007). However, the number of blocked embryos was higher in bscrFSH compared to the NIH-FSH-p protocol group (*p* = 0.035; Table 4). Finally, the number of unfertilised oocytes was not significantly different between SOV groups (*p* < 0.05).

### 3.5. Gene Expression in In Vivo Produced Bovine Embryos Obtained after NIH-FSH-p and bscrFSH-Derived SOV Protocol Application

**Figure 4:**
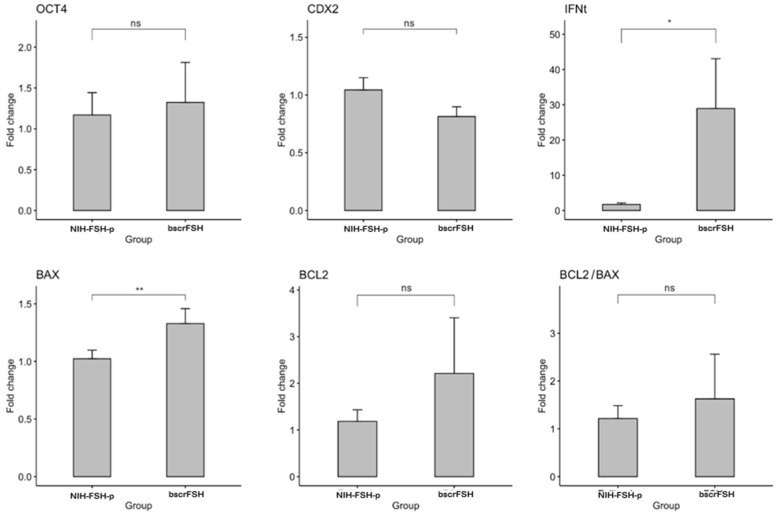
Gene expression analysis in in vivo produced bovine blastocysts by using a superovulation protocol that includes the standard pig pituitary-derived FSH (NIH-FSH-p) or extra-long-acting single-chain bovine follicle-stimulating hormone (bscrFSH). Bars represent the Fold Change in bscrFSH relative to NIH-FSH-p group. *, ** indicate significant differences between groups (*p* < 0.01 and *p* < 0.05, respectively). ns: non-significant.

Embryo quality was assessed by the analysis of the expression level of crucial genes for early embryo development (*OCT4, IFNt, CDX2, BCL2,* and *BAX*). Gene expression was analysed in individual embryos (blastocyst stage); 10 and 11 embryos from the NIH-FSH-p and bscrFSH treatments, respectively. *IFNt* and *BAX* were overexpressed in embryos from bscrFSH (*p* < 0.05) with a fold change of 16 and 1.3, respectively. However, no statistical differences were detected regarding *OCT4, CDX2, BCL2,* and the *BCL2/BAX* expression ratio (*p* > 0.05; Figure 4).

## 4. Discussion

Understanding the follicular dynamics and its self-regulatory mechanisms is crucial to study the basis for new alternatives to oestrous cycle control, follicular wave synchronisation and good-quality/competent bovine embryo production [4]. In the present study, an extra-long-acting single-chain bovine follicle-stimulating hormone (bscrFSH) has been synthesised by recombinant technology that includes additional N-glycosylation sites. The physiological functionality of bscrFSH has been evaluated by a single injection/day protocol (four decreasing doses) in order to superstimulate the ovarian follicles′ growth in beef cattle. The main objective of the present study was to evaluate the SOV responses as well as the embryo quality in cows treated with bscrFSH compared to others treated with a standard pig pituitary-derived FSH (NIH-FSH-p). The evaluation of the physiological effect of this new experimental bscrFSH showed a robust SOV response, taking into account the number of corpora lutea obtained, progesterone concentration levels, the number and quality of the embryos collected, the results of gene expression markers and fertility obtained when compared to SOV protocol by using conventional pituitary-derived FSH hormone (NIH-FSH-p).

Currently, the SOV treatments are still based on the use of pituitary-derived hormones in cattle. Although SOV protocols have been used over the last 40–50 years, the transferable embryos obtained per animal have not increased (around ~6 embryos/animal) [4,31]. In addition, SOV protocols using conventional FSH compounds present important problems such as the effects of other hormones on the expected results, variability within and between batches and the possibility of spreading diseases [31]. Consequently, several investigations have focused on the design of alternative methods to reduce the number and frequency of injections used in conventional SOV protocols [31,32], including once- daily subcutaneous (sc) injection of pituitary-derived FSH [33], once- daily sc FSH injection dissolved in saline or gelatin gel [34], a single intramuscular (im) FSH injection dissolved in polyvinylpyrrolidone [35], a single im FSH injection dissolved in hyaluronan [36], or even a single im FSH injection dissolved in hydroxide gel [37]. Despite all the protocols used with the aim to improve the SOV response in cattle, one of the main problems has been the variability in the population of gonadotropin-sensitive follicles at the time of SOV application and the lack of homology of the NIH-FSH-p molecule related to the receptors of bovine ovarian follicles [38]. In agreement with several studies using recombinant FSH, in the present research, reduced variation in gonadotropin-sensitive follicles has been observed [31,39]. It has also been described that the use of recombinant human FSH (rhFSH) without contaminating LH can elicit a normal superovulation response in bovine [40].

Although, during the last few years, recombinant gonadotropin strains have been designed and several studies on the use of rbFSH to induce SOV in heifers and cows have been reported, the results related to biological activity and SOV response have been variable, possibly due to the molecule being designed with fewer glycosylation groups [17,41]. Thus, during the last decade, a major effort to incorporate the use of recombinant bovine FSH and somatotropin has been carried out to develop more predictable SOV protocols compared to conventional FSH-p [42]. In the present study, promising results associated with the optimal SOV response and more viable and high-quality embryo rates have been obtained compared to other reported results by using conventional SOV protocols [25,43]. Consequently, it is necessary to point out that the use of extra-long-acting bscrFSH could be an interesting alternative to improve the results associated with the conventional SOV protocols.

The use of pure recombinant human FSH (rhFSH) in cattle without LH and equine chorionic gonadotropin (eCG) as a source of FSH with high LH activity has been described before, finding differences in oestradiol, progesterone and LH concentrations in SOV cows which could be related to the lack of LH activity together with a severe suppression of LH pulsatility [40]. Porcine follicle-stimulating hormone (FSH-p) and porcine luteinising hormone (LH-p) are widely used to induce superovulation in cattle. There is a variation in FSH:LH ratio among batches, affecting the follicular stimulation. Therefore, the relatively short FSH bioactivity (half-life) was compared to FSH-p, being 5 h and 10 to 12 h respectively, confirming the need for twice- daily injections [44,45]. SOV protocols have been modified and several molecules have been incorporated to improve the superovulation performance [38,42]. Thus, the use of recombinant bovine somatotropin (rBST) has been incorporated as a pre-treatment with conventional FSH-p in dairy cows, finding variability in the SOV response and the number and quality of transferable embryos [46,47]. Possibly, these results are due to the variability in commercial FSH and LH concentrations and their short half-life compared to others.

During the last few years, several molecules of FSH-free recombinant bovine follicle- stimulating hormone (rbFSH) have been developed. Baculovirus systems have been developed obtaining high production levels of bioactive rbFSH in bovine oocyte maturation inhibition assays and in rat Sertoli cells [48]. This synthesised hormone showed the free beta subunit as a doublet, probably indicating different glycosylated forms (MWs: 20 kDa (alpha subunit), 23 kDa (beta subunit), and 32.5 kDa (heterodimer)). These glycosidic side chains may not contain terminal sialic acid, which has possible negative implications for the glycosylation pattern, influencing the biological activity of the recombinant hormone [48]. Thus, the recombinant DNA-derived glycoprotein hormone rbFSH has been developed as an interesting alternative superovulatory agent in cattle. Several aspects, such as dose and duration, have been tested (doses of 0.5—12−24 mg twice/day over 3 to 5 days), with doses higher than 0.5 mg twice/day for 4 days being more efficient for producing embryos and a dose of 24 mg for 3 days being more effective than lower doses for the same duration [17]. Thus, it can be inferred that there is variability in the SOV response depending on the individual, possibly due to a lack of affinity for ovarian FSH receptors.

It has been reported that embryo production was not altered when individual cows were superovulated more than once at the selected dose, nor was subsequent reproductive performance affected [17]. Thus, the rbFSH hormone could elicit an SOV response without the need for exogenous LH, obtaining similar results to those reported previously by using pituitary extracts [17]. In contrast, several authors described that lower LH levels are necessary for follicular maturation and a better SOV response [49]. However, in the present study, the SOV response using LH-free bscrFSH was superior compared to other studies [50]. In the present research, the synthesised hormone dealt with a gene sequence design of a recombinant bovine single- chain FSH (bscrFSH) variant considering the alpha and beta chains of *Bos taurus* linked through a flexible spacer peptide with six potential N-glycosylation sites and an approximate protein MW ranging from 51 to 57 kDa depending on the glycoforms taken into account, obtaining an FSH purity level ≥ 97% and 0% of LH. These modifications are important because they increase both the stability and the half-life of the hormone after its application.

Regarding ovarian dynamics, the phenotypic selection based on follicle number may be useful to improve SOV procedures. Ovarian reserve size has been described to be positively associated with ovarian function and several fertility measures in *Bos taurus* cattle (e.g., progesterone secretion, endometrial thickness, response to superovulation, etc…) [51,52]. Several studies have investigated the effects of FSH dose on the response to SOV treatments [53]. Bó and Mapletoft [4], indicate that the ovulation rate increases as the FSH dose increases until a dose at which the ovulation rate eventually stabilises. Other authors have reported that the use of more than double the dose of FSH-p does not affect oocyte/embryo quality [54,55]. Even increasing the doses (double of the recommended) does not generate detrimental effects on fertilisation rates and the quality of transferable embryos [4]. However, in a recent review [56], it was described that a similar increase in the dose of crude pituitary extracts (containing both FSH and LH) resulted in significantly reduced percentages of fertilised oocytes and transferable embryos [54]. In addition, an excess of gonadotropins, mainly LH, would affect the quality of oocytes/embryos [56].

The authors of the present study agree that higher doses of FSH and LH could generate negative effects on SOV response, follicular development, oocyte competence and embryo quality due to the possible inactivation of the FSH follicle receptors, thus impairing the follicular development. Therefore, the use of pituitary-derived FSH with variable concentrations of FSH and LH could generate an important loss of homology related to FSH bovine follicle receptors compared to the use of bscrFSH, where a better SOV response was obtained regarding the number of functional corpora lutea. In another study [40], researchers compared the use of recombinant human FSH (rhFSH) without LH activity against the use of equine chorionic gonadotropin (eCG) in heifers and the same follicular growth and number of follicles (>8 mm in diameter) after 3 days of stimulation was observed. However, considerable differences in steroid production and final follicular maturation were observed being lower in heifers treated with rhFSH. This fact could be due to a lack of LH activity and a marked suppression of LH pulsatility in the study [40]. These results are in agreement with the present study mainly because the initial follicle growth of both bscrFSH and NIH-FSH-p was similar. However, a higher and more homogeneous final follicular growth was detected using bscrFSH compared to NIH-FSH-p maybe due to the greater homology of bscrFSH increasing the stimulation of follicle receptors and due to the fact that NIH-FSH-p has different LH concentration levels as well. Thus, the levels of exogenous LH could be detrimental according to the results described, as they could cause follicle luteinisation and/or atresia instead of follicular stimulation [56].

Carvalho et al. [18] compared several SOV protocols in heifers using a single injection of long-acting recombinant bovine FSH at different doses (medium and high) at a single application vs. medium FSH-p dose (eight applications, respectively). Differences in the number of ovulatory follicles have been observed, being higher for heifers treated with FSH-p and bscrFSH at eight medium- dose injections, and lower for heifers treated with single medium- and high- dose injections. Possibly, the use of medium doses of FSH-p or rbFSH is not sufficient to balance the FSH levels needed for follicle stimulation [18]. The results obtained in our study differed, as the number of corpora lutea was higher in cows treated with bscrFSH compared to NIH-FSH-p. This is probably because FSH-p and rbFSH have a short half-life compared to the extra-long half-life of bscrFSH used in our study. Thus, based on the results obtained in the present research, a single dose/day in a four day SOV protocol (four injections) was enough to generate SOV responses and a number of functional corpora lutea that was higher than the previous report, which also described that the number of good- quality embryos differed among treatments and was higher in heifers treated with high and medium rbFSH and FSH-p doses (eight injections) producing similar SOV responses and embryos compared to FSH-p (eight doses) [18]. In our trial a higher embryo number and quality were observed by using bscrFSH compared to NIH-FSH-p, possibly due to its higher homology.

Low progesterone concentrations and decreased ovarian follicular reserves (total number of oocytes/ovary) during reproductive cycles would be related to infertility in single ovulation species such as cattle. Results have shown that, despite of higher LH secretion progesterone concentrations are lower during oestrous cycles for individuals with low vs. high ovarian follicular reserves [51,52]. These individuals have also shown a decreased ability of granulosa cells to generate luteal cells and to produce progesterone. Therefore, it has been postulated that a high variation in ovarian follicular reserves in heifers and cows would be associated with alterations in optimal luteal differentiation and function (e.g., increased LH secretion and LH receptor desensitisation), decreasing LH responsiveness [52]. In beef heifers and lactating dairy cows it has been observed that progesterone concentrations between Day 3 and 14 of the oestrous cycle were 30% to 50% lower than in low versus high follicle count and such differences were repeatable from one oestrous cycle to the next [52]. Therefore, low serum progesterone concentrations would be associated with poor endometrial development, high embryo mortality [57] and infertility in cattle [58]. Therefore, embryonic mortality would be more frequent in cattle with low ovarian follicular reserves in relation to high reserves, higher numbers of functional corpora lutea and higher plasma progesterone levels. It has been described that there is a high similarity regarding the endometrial transcriptome on Day 9 in cows with high physiological progesterone compared to superovulated cows. It has been shown there are endometrial genes positively regulated by low progesterone concentrations involving the immune system and the endometrial inflammatory response. In contrast, cows with high physiological progesterone concentrations show a similar endometrial transcriptome profile to cows with good genetic merit for fertility, showing a positive gene regulation related to uterine relaxation- contraction, focal adhesion, GnRH signalling pathways and epidermal growth factors [59]. Therefore, it is suggested that a favourable endometrial environment for embryo survival and development would be contingent on higher progesterone concentrations associated with a higher number of functional corpora lutea and ovarian follicular reserve.

In the present study, bscrFSH SOV cows presented higher levels of plasma progesterone as a result of a higher number of corpora lutea compared to cows superovulated by using NIH-FSH-p. It has been reported that serum progesterone concentrations on Day 7 (embryo collection/Day 15) could differ depending on the dose of NIH-FSH-p together with the addition of nutritional supplements in cows [60]. This fact would be related to improved follicular development and corpus luteum formation and functionality; however, it is important to consider that factors such as FSH origin and ovarian follicular reserve are directly associated with the obtained results. Thus, progesterone concentrations possibly were not influenced by the treatment itself, but could have been affected by treatment interactions. Progesterone decreased to basal levels earlier in heifers treated with FSH-p than in heifers treated with Human Menopausal Gonadotropin (HMG) [61].

With regard to serum cortisol concentrations when using medium and high FSH-p doses, higher cortisol levels have been observed when using high FSH-p doses [60]. However, cortisol concentrations on the day of embryo collection were lower, and, there were higher concentrations at the start of protocol on Day 0, at the onset of oestrus and at the first day of insemination [60]. In cattle, the confinement, human- animal interactions and treatment administrations may cause an acute stress response in the donors showing marked effects on embryo viability. Thus, stress has been associated with a reduced SOV response and embryo quality [61,62]. The suppression of reproductive function during stress is due to the negative feedback of cortisol on the hypothalamic- pituitary axis, resulting in a decrease in LH pulse rate [63]. A decrease in LH pulse rate suppresses follicular growth, ovulation rate and fertilisation [63]. However, it has been described that local ovulation induces a follicular inflammatory response [64], which would increase with a greater number of preovulatory follicles after ovulation [65]. This increase in preovulatory follicle wall cortisol levels is induced by an increase in LH levels [66]. Thus, the preovulatory follicle would activate the cortisol/LH mechanism for the regulation of the inflammatory reactions associated with ovulation. Although cortisol levels in superovulated heifers during the oestrus reach up to 20.6 ng/mL [65], it would not be detrimental to the oocyte, and consequently to the embryo.

Regarding the SOV protocols used in the present study, taking into account the total viable grade I embryos obtained significantly differences have been observed between bscrFSH and NIH-FSH-p. Other reported studies showed no differences among treatments, possibly because the recombinant FSH used had a shorter half-life and was very similar to pituitary-derived FSH [7]. The recovery of oocyte/embryonic structures (non-viable embryos, degenerated embryos and unfertilised oocytes) was comparable to previous studies that used FSH-p during the SOV protocols [67]. However, in the present research the bscrFSH half-life was longer and this fact could explain the different results obtained. Thus, in the present research the number of viable embryos was higher in cows treated with bscrFSH compared to the NIH-FSH-p SOV protocol. Although no differences were observed regarding the number of embryos recovered in further stages than morula, there were statistical differences in the number of morulae obtained between protocols. Taking into account that the morula stage has been considered in the scientific literature as an early form of a transferable embryonic stage, the efficiency was improved by using bscrFSH in terms of the number of total transferable embryos obtained. These results by using bscrFSH were consistent and, thus, bscrFSH could be considered as an interesting alternative for use in SOV protocols in the early future. Regarding the morula stage, as stated before, it can be considered as the early form of transferable embryos [68]. In our study the treatment with bscrFSH showed significant differences regarding morula rates assuming that bscrFSH would have a higher affinity for follicle receptors. Therefore, in the follicular recruitment and selection stage, the bscrFSH supply could be more homogeneous and constant, leading to better follicular superstimulation. In this context, pre-ovulatory follicles would have a narrower and more synchronous ovulation range, resulting in a more concentrated ovulation in a shorter time, in contrast to pituitary FSH where the ovulation is progressive over time. Furthermore, the transfer of embryos at different stages of development (morula and blastocyst) has no effect on the pregnancy rate in beef heifers [69]. Therefore, if a higher percentage of morulae per donor is obtained it is assumed that the stimulation and the ovulation synchronisation should be more successful. This could demonstrate a better effects due to the higher homogeneity (purity) of the bscrFSH compared to the higher variability observed and reported with the pituitary-derived FSH [18]. Due to the purity grade of bscrFSH compared to NIH-FSH-p, together with the absence of LH in the first one, the total dose adjustment for both protocols was different. In previous pilot assays, the total dose was adjusted in order to avoid an overstimulation due to the bscrFSH purity grade. Moreover, slightly different doses of bscrFSH or slight adjustments in the timing of procedures, e.g., modifications in the timing of CIDR removal or even an increase from a 4-day FSH to a 5-day FSH SOV protocol or even more, might result in a higher yield of blastocysts in comparison to morulae [56,70].

Several studies have been published, indicating that the lower proportion of transferable embryos after SOV in ovaries with a large number of follicles was likely not due to differences in oocyte quality [51]. Taking this fact into account, several biomarkers related to ovarian reserves, ovarian function and fertility have been studied in cattle [71]. In this regard, it has been described that anti-Müllerian hormone (AMH) concentrations are static within individuals and correlate positively with ovarian follicular populations; however, fertility rates and circulating AMH concentration were not correlated in heifers and cows with low AMH [72]. Therefore, it is inferred that AMH and follicle- stimulating hormone receptor (FSHR) mRNA expression prior to SOV may predict the positive response of donor cows to SOV [73]. This fact is important to consider when selecting donors before initiating a SOV protocol, although positive outcomes are also dependent on other factors, including the FSH origin.

The quality and competence of produced embryos is also of great concern when a SOV protocols are applied. Embryo quality is positively correlated with implantation and pregnancy maintenance. Besides embryo morphology, the gene expression pattern is correlated with embryo competence [74]. The control of cellular potency, differentiation, and death, as well as trophoblast function, is considered as crucial factor during early embryo development [75,76]. In this regard, the expression patterns of genes that are involved in or control these pathways are used as indicators of embryo quality and competence [77,78]. In this work, the expression levels of *OCT4*, *IFNt*, *CDX2*, *BCL2,* and *BAX* were compared between embryos produced in vivo by using a superovulation protocol that included NIH-FSH-p or bscrFSH. The expression of *NANOG*, *SOX2* and *FGF4* was detected in some but not all embryos despite the experimental group. This could be explained by the time difference in the events that take place during cell lineage segregation in each evaluated embryo and that are not morphologically evident at the blastocyst stage [77,79,80]. *BAX*, a proapoptotic gene, was over expressed in embryos from the bscrFSH group. After embryonic genome activation (*EGA*), embryos show signs of apoptosis, including DNA fragmentation and expression of *BAX* [81]. However, the activation of apoptosis in early embryos might be a mechanism to keep a balance between cell death and proliferation despite the presence of a stressor or oocyte quality [82]. The increase in the expression of *BAX* has been associated with the poor morphology of the embryos [83]. However, in the current work, no morphological differences were observed among embryos from the different superovulation protocols (data not shown). Moreover, no statistical differences were observed for *BCL2*, an anti-apoptotic gene, and the ratio of *BCL2*/*BAX* between the groups. It is suggested that the ratio between pro- and anti-apoptotic genes is a better marker of bovine embryo quality than the expression levels of individual genes [84].

Interferon tau (IFNt) is the main signal for pregnancy recognition in bovine. Its expression starts at the time of blastocoel formation and its secretion increases during the elongation period [42,85]. A correlation between IFNt secretion and the blastocyst morphological quality has not been proven, but it is positively correlated with conceptus length at the time of elongation [86]. The role of IFNt remains controversial. It has been reported that there is a negative relationship between early IFNt production and embryo competence [87]. On the other hand, a positive correlation has been observed between IFNt production and early embryo competence in both in-vitro and in-vivo-produced embryos in bovine [88]. It is important to note that gene expression (including *INFt*) in *in- vivo*- produced embryos when using ovarian hormonal superstimulation treatments could alter the embryo gene expression in bovine [89]. In fact, studies in other mammals have shown that ovarian hormonal stimulation may affect the development and the embryonic gene expression in many different ways [90,91]. Taking into account the effects of ovarian hormonal stimulation on the embryonic gene expression reported before, the increase in the *INFt* expression level obtained from the bscrFSH-derived compared to the NIH-FH-p-derived SOV protocol may be related to the use of the extra-long-acting bscrFSH (97% purity) used in the present study. As shown in the results, bscrFSH induced higher ovarian superstimulation effects, which may explain, at least in part, the differences obtained with regard to *IFNt* expression between SOV protocols, even when no embryonic morphological differences were observed. In fact, the role of IFNt during early pregnancy is determined by different factors, including both ovarian function and embryonic development, as long as the IFNt plays a critical role in early pregnancy due to its antiluteolytic effects [92,93].

## 5. Conclusions

Including bscrFSH in SOV protocols would be an important alternative by reducing the number of applications and offering a better SOV response, together with the efficient production of viable embryos for fresh transfer or cryopreservation purposes. Therefore, according to the results obtained, the administration of a single dose/day at 24 h intervals (four- application SOV protocol) offers a new, safe and efficient alternative compared to the conventional pituitary-derived FSH (NIH-FSH-p) used in SOV protocols in bovine. The reduction in the number of FSH applications has the advantage of reducing animal welfare problems and labour resources and obtaining better results compared to commercial pituitary-derived FSH extracts. Further studies using different cattle breeds are needed to compare the effects of bscrFSH under different conditions, such as individual sensitivity, species and the optimisation of SOV doses, also taking into account the age of the individuals studied.

## Figures and Tables

**Table 1:** Primers used for PCR and sequencing in the present study.

Gene	Primer Forward Sequence (5′-3′)	Primer Reverse Sequence (5′-3′)	Amplicon Size (bp)	Annealing Temp. (°C)
**OCT4**	TCGAGAACCGAGTGAGAGGC	ACACTCGGACCACGTCTTT	120	55
**BAX**	CGGGTTGTCGCCCTTTTCTAC	CAGCCGCTCTCGAAGGAAGT	120	55
**CDX2**	AAACCCTACTGTCACCCAGT	TGAGGGTTCTAGCAGAGTCCA	90	56
**INFt**	ATGCTCCAGCAGTGCCTCAAC	TGTTGGAGCCCAGTGCAGAG	95	57
**BCL2**	TGTGGAGCTGTATGGCCCTAGC	AGATAGGCACCCAGGGTGATGC	114	56
**ACTB**	TGCCCTGAGGCTCTCTTCCA	TTGGCGTAGAGGTCCTTGCG	119	55
**GAPDH**	AGGTCGGAGTGAACGGATTC	ATGGCGACGATGTCCACTTT	85	56

*OCT4:* octamer-binding transcription factor 4; *BAX: BCL2* Associated X, anti-or pro-apoptotic regulators; *CDX2:* homeobox transcription factor; *INFt:* interferon tau; *BCL2:* B-cell lymphoma 2, apoptosis regulator; *ACTB:* actin beta; *GAPDH:* glyceraldehyde-3-phosphate dehydrogenase; *ACTB* and *GAPDH* genes were used as housekeeping controls.

**Table 2:** Ovarian dimensional evaluation over time: NIH-FSH-p vs. bscrFSH SOV protocol.

Time Points		Day 0		Day 4		Day 8		Day 15	
SOV		NIH-FSH-p	bscrFSH	NIH-FSH-p	bscrFSH	NIH-FSH-p	bscrFSH	NIH-FSH-p	bscrFSH
Protocol									
**Ovarian Dimensions**	**Length**	2.32 ± 0.07 ^a^	2.73 ± 0.08 ^a^	2.28 ± 0.05 ^a^	2.79 ± 0.07 ^a^	4.71 ± 0.11 ^b^	5.10 ± 0.17 ^b^	9.39 ± 0.16 ^c^	9.41 ± 0.18 ^c^
	**Width**	1.74 ± 0.07 ^a^	1.83 ± 0.08 ^a^	1.61 ± 0.05 ^a^	1.77 ± 0.07 ^a^	4.28 ± 0.12 ^b^	4.86 ± 0.21 ^b^	8.73 ± 0.49 ^c^	8.94 ± 0.24 ^c^

Values (Mean ± S.E.M.) within a row with different superscript letters (a–c) between time-points are significantly different (*p* < 0.05). No differences were observed regarding ovarian length or width within the same time-point (day) between both SOV treatments (*p* > 0.05). Unit: cm.

**Table 3:** Hormone level profiles (progesterone (P4) and cortisol (C)) over time: NIH-FSH-p vs. bscrFSH SOV protocol.

Time Points		Day 0		Day 4		Day 8		Day 15	
SOV		NIH-FSH-p	bscrFSH	NIH-FSH-p	bscrFSH	NIH-FSH-p	bscrFSH	NIH-FSH-p	bscrFSH
Protocol									
**Hormone Levels**	**P4**	4.14 ± 0.26 ^a^	3.85 ± 0.30 ^a^	5.21 ± 0.29 ^a^	6.07 ± 0.52 ^a^	1.00 ± 0.08 ^b^	0.92 ± 0.09 ^b^	61.27 ± 7.35 ^cA^	74.13 ± 8.93 ^cB^
	**C**	3.43 ± 0.20 ^a^	4.13 ± 0.23 ^a^	4.67 ± 0.24 ^a^	6.05 ± 0.52 ^a^	13.14 ± 0.84 ^b^	12.51 ± 0.67 ^b^	6.27 ± 0.37 ^a^	5.87 ± 0.41 ^a^

Values (Mean ± S.E.M.) within a row with different superscript letters (^a–c^) between time points (days) are significantly different (*p* < 0.05). Values (Mean ± S.E.M.) within a row with different superscript letters (^A,B^) within each time-point (day) are significantly different (*p* < 0.05). Unit: ng/mL.

**Table 4:** In vivo produced bovine embryos by using different superovulation (SOV) protocols including the standard pig pituitary-derived FSH (NIH-FSH-p) and the extra-long-acting single-chain bovine follicle-stimulating hormone (bscrFSH).

In Vivo Embryo Production Parameters	SOV Protocols	
	NIH-FSH-p (Mean ± SEM)	bscrFSH (Mean ± SEM)
Total Number of Structures (TNS)	8.00 ± 0.60 ^A^	10.32 ± 0.81 ^B^
Unfertilized Oocytes (UFOs)	0.50 ± 0.13 ^A^	0.71 ± 0.26 ^A^
Morulae (M)	2.85 ± 0.55 ^A^	5.47 ± 0.75 ^B^
Early Blastocysts (EB)	1.79 ± 0.31 ^A^	1.32 ± 0.27 ^A^
Blastocysts (B)	1.65 ± 0.30 ^A^	1.88 ± 0.54 ^A^
Viable Embryos (VE)	6.32 ± 0.56 ^A^	8.65 ± 0.67 ^B^
Degenerated Embryos (DE)	1.29 ± 0.25 ^A^	0.44 ± 0.16 ^B^
Blocked Embryos (BE)	0.12 ± 0.07 ^A^	0.47 ± 0.14 ^B^

Different letters within the same row (A and B) represent statistical differences between SOV protocol groups (*p* < 0.05).

## Data Availability

All data generated or analyzed during this study are included in this article.
